# Leveraging deep learning to infer continuous predictions from ordinal labels in medical imaging

**DOI:** 10.1371/journal.pdig.0001248

**Published:** 2026-04-17

**Authors:** Katharina V. Hoebel, Andréanne Lemay, John Peter Campbell, Susan Ostmo, Michael F. Chiang, Christopher P. Bridge, Matthew D. Li, Praveer Singh, Aaron S. Coyner, Jayashree Kalpathy-Cramer

**Affiliations:** 1 Martinos Center for Biomedical Imaging, Boston, Massachusetts, United States of America; 2 Harvard-MIT Division of Health Sciences and Technology, Massachusetts Institute of Technology, Cambridge, Massachusetts, United States of America; 3 NeuroPoly, Polytechnique Montreal, Montreal, Quebec, Canada; 4 Oregon Health and Science University, Portland, Oregon, United States of America; 5 National Eye Institute, National Institutes of Health, Bethesda, Maryland, United States of America; 6 National Library of Medicine, National Institutes of Health, Bethesda, Maryland, United States of America; 7 MGH & BWH Center for Clinical Data Science, Boston, Massachusetts, United States of America; 8 University of Colorado Anschutz Medical Campus, Aurora, Colorado, United States of America; University of Cambridge, UNITED KINGDOM OF GREAT BRITAIN AND NORTHERN IRELAND

## Abstract

In clinical medicine, variables like disease severity are often categorized into discrete ordinal labels such as normal/mild/moderate/severe. However, these labels, commonly used to train and evaluate disease severity prediction models, simplify an underlying continuous severity spectrum. Using continuous scores can aid in detecting small severity changes more sensitively over time. We introduce a deep learning based approach that predicts continuously valued variables from medical images using only discrete ordinal labels during model development. We evaluated this approach using three medical imaging datasets: disease severity prediction for retinopathy of prematurity and knee osteoarthritis, and breast density prediction from mammograms. Deep learning models were trained with discrete labels, and model outputs were transformed into continuous scores. These were then compared against detailed expert severity assessments, which exceeded the granularity of training labels. Our study explored conventional and Monte Carlo dropout multi-class classification, ordinal classification, regression, and twin models. We found that models incorporating the ordinal nature of training labels significantly outperformed conventional multi-class classification. Notably, continuous scores from ordinal classification and regression models demonstrated a higher correlation with expert severity rankings and lower mean squared errors than multi-class models. The application of Monte Carlo dropout further enhanced the prediction accuracy of continuously valued scores, aligning closely with the continuous target variable. Our findings confirm that accurate continuous scores can be learned from discrete ordinal labels using deep learning, offering a robust method that effectively bridges the gap between discrete and continuous data across various image analysis tasks.

## Author summary

Physicians often describe disease severity using categories like mild, moderate, or severe. However, disease severity exists on a continuous scale, with small differences that the commonly used broad categories cannot capture. This can make it harder to track changes over time. Our study systematically assesses how deep learning models can be trained to predict more precise scores for disease severity from images, even when they are trained using only simple discrete severity labels. We tested our approach on three distinct prediction tasks in medical imaging: retinopathy of prematurity, knee osteoarthritis, and breast density. We found that models that respect the inherent ordinal structure of the training labels generate more precise continuous scores, closely aligning with expert assessments. Furthermore, incorporating Monte Carlo dropout further improved the accuracy of these predictions. In summary, our findings show that the gap between categorical ordinal labels and the continuous nature of disease progression can be closed, enabling more sensitive assessments of disease severity. Ultimately, providing more detailed automatic assessments of disease severity could improve clinical decision-making by allowing earlier detection of disease deterioration and more personalized treatment planning.

## 1 Introduction

**Fig 1:**
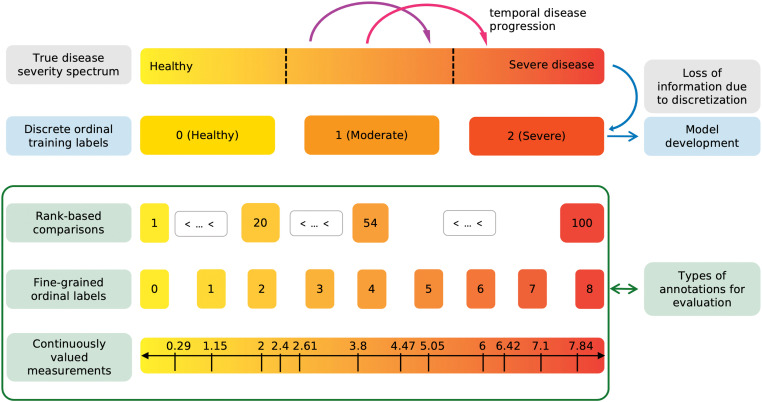
Relationship between a continuously distributed disease severity variable and the labels used for training and evaluation. Discretization of the true disease severity spectrum into discrete ordinal training labels results in an inherent loss of information. The purple and magenta arrows depict temporal disease progression, which may go undetected when using ordinal labels (purple arrow). For model evaluation, we leverage more granular annotations, including rank-based comparisons, fine-grained ordinal labels and continuously valued measurements (green box). These finer-scale labels enable a more nuanced assessment of model performance in capturing the underlying continuous nature of disease severity.

Many clinical variables, like disease severity, are communicated and recorded as discrete ordinal classes. However, in reality, they are distributed on a continuous spectrum [1]. The discretization of continuously valued variables facilitates documentation and communication and standardizes treatment decisions at the cost of losing information. As illustrated in Fig 1, two patients that fall into the same severity category will receive the same label. Consequently, if their data is used to develop a prediction model, it will be treated exactly the same during training, regardless of their exact position along the continuous spectrum.

The ability of deep learning (DL) to learn and recognize subtle patterns from large amounts of data has led to tremendous successes in automated medical image analysis. [2] DL models have achieved and, in some cases, even exceeded human performance in disease detection and automatic severity classification for numerous diseases such as diabetic retinopathy, retinopathy of prematurity (ROP), and osteoarthritis [3–5].

Yet, these successes have been built on the simplifying assumption that disease severity prediction can be formulated as a simple classification task. Moreover, researchers mostly use DL architectures that are intended for the classification of nominal categories and ignore the inherent ordinal nature of the available training labels. Additionally, disease severity prediction tasks are often simplified even more by treating them as binary problems, e.g., the identification of severe disease, cases for referral, or disease detection [3,6–8].

**Advantages of continuous scores.** In addition to the class, the position of a case on a continuous spectrum of, e.g., disease severity, contains valuable clinical information that is not captured by current approaches [9]. Therefore, using continuous scores to describe clinical variables distributed on a continuous spectrum provides several advantages over discrete ordinal variables.

First, continuous metrics allow for the detection and quantification of changes within a class, i.e., an increase in disease severity that does not constitute a transition between class *n* and n+1 [10,11]. In Fig 1 the purple and magenta arrows represent similar large increases in disease severity. While the magenta transition would be detected by the traditional classification approach, the purple transition would not. The only difference between the two transitions is that the magenta one crosses the class boundary from moderate to severe, while the purple arrow represents a within class change. The detection of within-class changes allows us to bring disease deterioration earlier to the attention of the care team and act upon it if required.

Second, the higher degree of information presented in continuous versus ordinal scores can be useful for efficient patient stratification, particularly the identification of cases close to a decision boundary. Third, expert perception of class boundaries can be subject to changes over time [12]. Therefore, models ignoring the continuous nature of disease severity could become less valuable over time as, e.g., the perception of what constitutes mild versus moderate disease severity shifts. Lastly, an algorithm that predicts a continuous score is more likely to fulfill notions of individual fairness as similar individuals that are close to the label decision boundaries will be more likely to receive similar scores compared to using a simple classification algorithm [13].

**Related work.** Ordinal classification has been extensively studied, particularly in applications such as sentiment analysis, credit scoring, and disease severity prediction [14–16]. Deep learning-based ordinal regression has been applied in medical imaging to improve classification performance, with studies exploring frameworks for applications such as breast density prediction and chest radiographs [17,18]. Recently proposed methodological advances include integrating convolutional neural networks with differential forests and enhancing robustness in brain age prediction through ordinal-distance regularization, and the introduction of ordinal conformal prediction sets to enhance the trustworthiness of DL-based severity ratings [19–21].

Despite advancements, these approaches remain limited to predicting discrete ordinal labels and do not attempt to bridge the gap between the underlying continuous variables and their ordinal representations, which is crucial for capturing finer variations in the underlying prediction target. To the best of our knowledge, only two prior studies have attempted to predict continuous disease severity scores from ordinal labels using conventional classification networks or twin networks [10,22]. Redd et al. proposed to aggregate the softmax outputs from a conventional 3-class convolutional neural network into one continuously valued vascular severity score for disease severity classification in ROP [22]. This score strongly correlates with experts’ ranking of overall disease severity. Furthermore, changes in this score over time accurately reflect disease progression [11]. However, the training objective of multi-class classification models is to separate the latent space representation of classes as much as possible. This could, therefore, lead to unstable predictions and confusion at the class boundaries.

Using twin networks, Li et al. showed that the continuously valued difference relative to a reference pool of images correlates with expert’s disease severity rankings and reflects temporal severity changes in knee osteoarthritis and ROP [10]. Similarly, in a study on an x-ray based severity score for COVID-19, a score generated by a twin network highly correlated with radiologist-determined severity scores. However, the performance of twin networks for the predictions of continuous scores has not been compared to other methods, and their calibration has not yet been studied.

Monte Carlo (MC) dropout, originally introduced as a Bayesian approximation technique, provides uncertainty estimates by applying dropout at inference time and averaging multiple stochastic forward passes [23]. In the context of ordinal classification, MC dropout can improve model calibration and enhance the repeatability of predictions [24]. Given that our objective is to recover the continuous nature of the target variable from discrete ordinal labels, we hypothesized that improved calibration via MC dropout could contribute to more stable and meaningful continuous severity predictions. However, while uncertainty modeling techniques have been widely explored in other domains, their impact on continuous severity scoring remains underexplored.

**Study aim.** Here, we extend prior work by identifying model development strategies for convolutional neural networks that lead to the prediction of accurate continuous scores from medical images. Most importantly, while we utilize widely available discrete ordinal labels for training, the models’ performance to predict accurate continuous scores is evaluated using labels on a finer scale than the training ground truth (illustrated in the green box in Fig 1) on three datasets: disease severity prediction for ROP and knee osteoarthritis, from retinal photographs and knee radiographs, respectively, and breast density estimation from mammograms. Following this process, we aim to show that it is possible to develop models that are capable to recover the information lost through the discretization of the continuous target variable.

## 2 Materials and methods

### 2.1 Datasets

We evaluated our approach across three different medical imaging prediction tasks, each involving the estimation of a continuous severity-related score from ordinal labels: Predicting ROP disease severity prediction from retinal photographs, knee osteoarthritis severity from knee radiographs, and breast density from mammograms. All images were de-identified prior to data access; ethical approval for this study was therefore not required. Dataset splits were performed on a patient level. The size of all dasets is listed in Table 1 and class distributions for each dataset are listed in S1 Text.

**Table 1:** Summary of dataset size and training/validation/test splits of the three datasets used for this study: disease severity prediction in retinopathy of prematurity (ROP) and knee osteoarthritis (OA) and breast density prediction.

Dataset	Size	Training	Validation	Test
ROP	5611	4322	722	467 (9-point scale)
				100 (ranked)
Knee OA	14273	12268	1905	100 (ranked)
Breast Density	83034	70293	10849	1892 (Volpara Density)

#### 2.1.1 Retinopathy of prematurity.

ROP is an eye disorder mainly developed by prematurely born babies and is among the leading causes of preventable childhood blindness [25]. It is characterized by a continuous spectrum of abnormal growth of retinal blood vessels which is typically categorized into three discrete severity classes: normal, pre-plus, or plus [1,26]. We use the same images, labels, and preprocessing as described by Brown et al. [4]. In addition to the standard diagnostic labels, the test set was labeled by five raters on a scale from 1 to 9, where a score of 1 corresponds to a normal appearance of the retina and a score of 9 represents maximal vascular abnormalities. Furthermore, five experts ranked an additional 100 ROP photographs based on severity [4,27].

#### 2.1.2 Knee osteoarthritis.

The global prevalence of knee osteoarthritis is 22.9% for individuals over 40, causing chronic pain and functional disability [28]. Knee osteoarthritis can be diagnosed with radiographic images and disease severity is typically evaluated using the Kellgren-Lawrence (KL) scale consisting of the following severity categories: none, doubtful, mild, moderate, and severe [29].

We use the the Multicenter Osteoarthritis Study (MOST) dataset. 100 images from the test set were ranked by their severity by three experts [10]. All images were center-cropped to 224x224 pixels and intensity scaled between 0 and 1 as preprocessing.

#### 2.1.3 Breast density.

Breast density is typically categorized as fatty, scattered, heterogeneous, or dense, depending on the amount of fibroglandular tissue present [30]. Women with high breast density are at a higher risk of developing breast cancer and require additional MRI screening [31,32]. We use a subset of the Digital Mammographic Imaging Screening Trial (DMIST) dataset [33]. Categorical breast density labels based on expert radiologists’ visual assessment were used as ground truth for model development. For evaluation, we used a subset of 1892 images from the test set where an automatic assessment of the continuously valued volumetric breast density was obtained using the commercially available Volpara Density software (Volpara Imaging Software 1.5.11; Mātakina, Wellington, New Zealand) [34]. The Volpara Density measurements were used solely for post-hoc evaluation. Since the Volpara algorithm applies a relative physics model to determine volumetric breast density based on mammographs, its measurements serve as an independent proxy for breast density that is distinct from both the expert-derived training labels and DL model predictions. Measurements of breast density acquired using this software have previously demonstrated a good agreement with expert ratings (see Fig 5) [34,35]. Preprocessed mammograms were of size 224x224 pixels.

**Fig 2:**
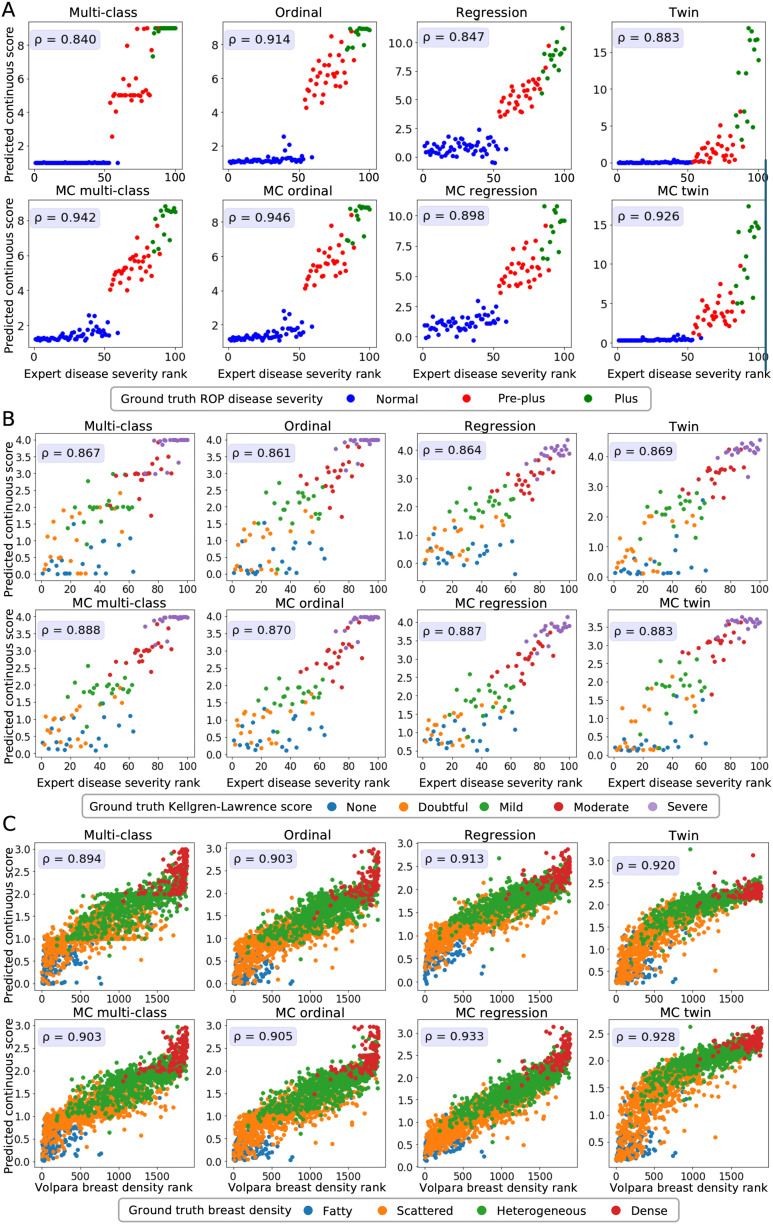
Relationship between predicted continuous scores, expert rankings, and discrete ordinal ground truth labels across multiple datasets. A: Retinopathy of prematurity (ROP) disease severity prediction; B: Knee osteoarthritis severity prediction; C: Breast density prediction. Each subplot represents a different model type (multi-class, ordinal, regression, twin) without (top rows) and with Monte Carlo (MC) dropout (bottom rows in panels A, B, and C). For each model, the Spearman correlation coefficient (*ρ*), displayed in the upper left corner, quantifies how well the predicted scores preserve the expert-derived (A, B) or Volpara Density-derived rankings (C). Higher *ρ* values indicate stronger alignment.

**Fig 3:**
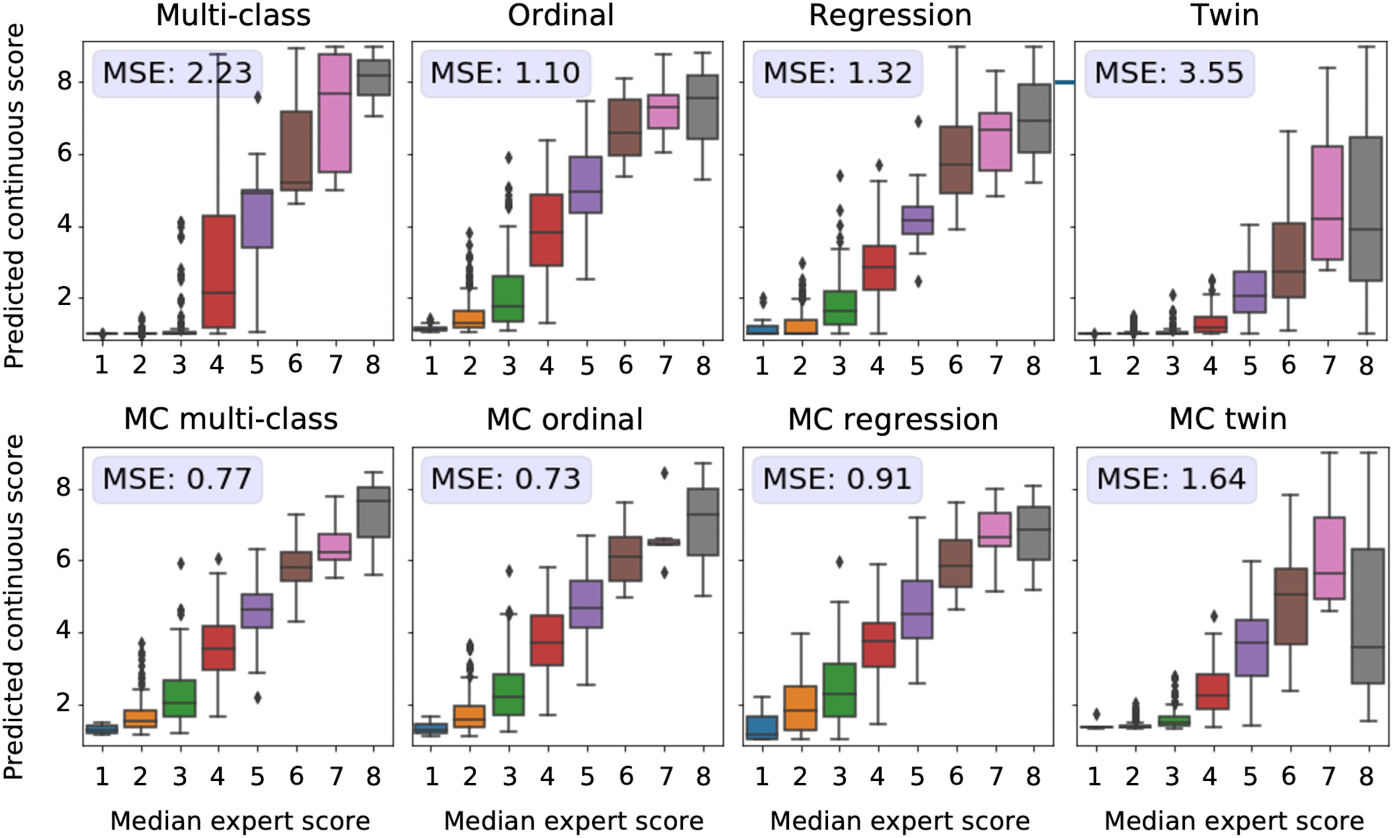
Relationship between predicted and expert-derived severity scores for Retinopathy of prematurity (ROP). Each subplot represents a different model type (multi-class, ordinal, regression, twin) without (top row) and with Monte Carlo (MC) dropout (bottom row). The x-axis represents the median severity score assigned by five expert raters on a scale from 1 to 9, while the y-axis shows the distribution of the predicted continuous scores. The predicted scores from multi-class, ordinal, and regression models that were trained to predict values between 0 and 2 were linearly transformed to match the 1 to 9 range ($score_{\text{rescaled}}=(score_{\text{model}}\times 3)+1$). Predictions obtained from twin networks that predict unbound similarity scores were shifted and clipped to match the 1-9 scale ($score_{\text{rescaled}}=score_{\text{twin}}+1$). Mean squared error (MSE) values quantify the prediction error for each model, with lower MSE indicating better alignment with expert ratings. All MSE measurements reported in this figure are statistically different (p-value <1.2e−41), indicating meaningful differences in prediction performance across models.

**Fig 4:**
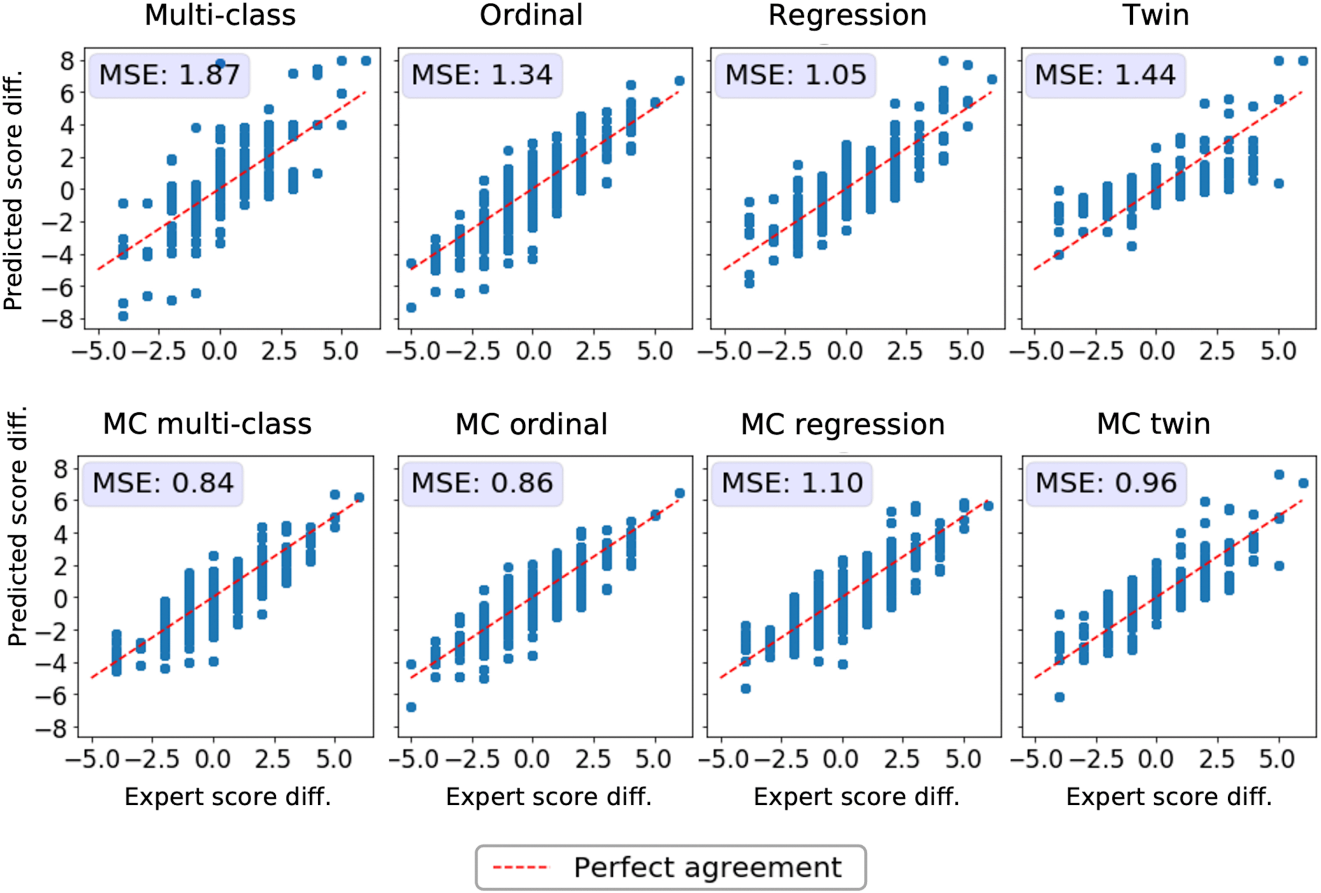
Agreement between predicted and expert-rated severity changes over time. Each subplot represents a different model type (multi-class, ordinal, regression, twin) without (top row) and with Monte Carlo (MC) dropout (bottom row). Disease severity changes for ROP patients were calculated based on differences in severity assessments between two images of the same patient acquired at different time points. The x-axis represents that change in the expert-derived severity scores and the y-axis the change in model predictions. The red dashed identity line represents perfect agreement, where predicted differences align exactly with expert-assessed differences. Means squared error (MSE) values indicate the degree of prediction error, with lower values signifying better alignment. Differences between all reported MSE values are statistically significant (p−value<4.3e−28).

**Fig 5:**
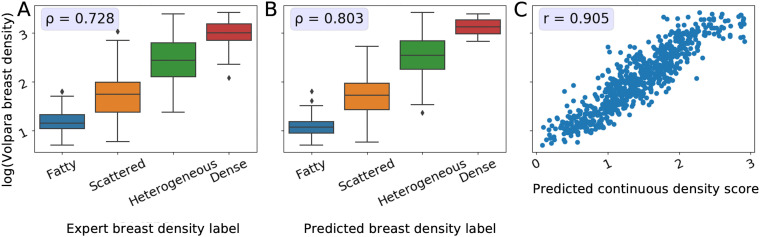
Relationship between the continuous Volpara Density measurements and expert- and model-derived breast density predictions. A: Distribution of the log-transformed Volpara Density measurements across the discrete ordinal expert-labeled breast density categories used for model development; B: Distribution of the log-transformed Volpara Density measurements across predicted breast density categories; C: Agreement between the log-transformed Volpara Density measurements and predicted continuous breast density scores. The predicted values are generated using the Monte Carlo multi-class model. Spearman’s correlation coefficient, *ρ*, indicates how well the categorical labels preserve the ordinal structure of the Volpara measurements (A and B) and Pearson’s correlation coefficient, *r*, measures the linear relationship between the log-transformed Volpara measurements and the predicted continuous breast density scores (C).

### 2.2 Model types

To identify the model type best suited to obtain accurate, continuously valued predictions, we trained four model types: multi-class, as used by Redd et al. [22] and ordinal classification, regression, and twin, as used by Li et al. [10]. The model output was converted to a continuous score value for each model to represent the underlying severity spectrum of the medical task.

**Classification.** We compute continuous severity scores based on predictions of nominal classification models as the weighted sum of the softmax outputs (Eq 1), resulting in scores ranging from 0 to K−1.


$$Cl_{score}=\sum_{i=1}^{K}p_{i}\times i-1,$$ (1)

with *K* being the number of classes and $p_{i}$ the softmax probability of class *i*. All classification models for this study were trained with cross-entropy loss.

**Ordinal classification.** In ordinal classification, the ordinal multi-class classification task is broken down into K−1 binary classification tasks. Therefore, the output layer consists of K−1 neurons [36,37]. During training, the ordinal loss function (Eq 2) penalizes larger misclassification errors more than smaller errors (e.g., predicting class 2 when the ground truth label is 0 is penalized more than if the models predict class 1). We use the CORAL loss as described by Cao et al. for model optimization [39].


$${ℒ}_{ordinal}=-\sum_{k=1}^{K-1}[y_{\leq k}log\sigma ({\hat{y}}_{k})+(1-y_{\leq k})log(1-\sigma ({\hat{y}}_{k}))]$$ (2)

where *K* is the total number of ordinal categories, $\sigma ({\hat{y}}_{k})$ represents the sigmoid-transformed logit for category k, and $y_{\leq k}$ is a binary indicator that is 1 if the true label y is less than or equal to category *k*, and 0 otherwise.

A continuous score is generated by summing over the output probabilities (Eq 3), resulting in values ranging from 0 to K−1.


$$O_{score}=\sum_{i=1}^{K}p_{i}.$$ (3)

**Regression.** Similar to ordinal models, regression models require the ordinality of the target output. However, unlike ordinal models, the output of regression models is a continuous value rather than a discrete class. The regression models were trained using mean squared error loss with integer-valued class labels as the target values. The raw model output yields a continuous value; hence, no conversion is required to receive a continuous score.

**Twin networks.** Twin networks compare pairs of images to evaluate their similarity [38]. They are composed of two branches consisting of identical sub-networks with shared weights where each of the two images is processed in one of the branches. The lower the Euclidean distance between the outputs of each branch the higher the similarity between the inputs. Following a procedure described by Li et al., at test time, we obtain predictions of the similiarity between the target image and a pool of ten anchor images representing class 0 [10]. Here, the continuous score is the median of the Euclidean distances between the target and the ten anchor images.

### 2.3 Monte Carlo dropout

To quantify predictive uncertainty, we employed Monte Carlo (MC) Dropout, which serves as a computationally efficient approximation to Bayesian Neural Networks [23]. While standard dropout is typically deactivated during inference, MC Dropout retains stochasticity at test time. By performing *N* stochastic forward passes, we effectively drew samples from a learned approximate posterior distribution over the network weights. This process can be viewed as querying an ensemble of slightly different model configurations, where the variance among the *N* predictions reflects the model’s epistemic uncertainty.

In this study, the resulting MC predictions were averaged to obtain the final output. All models referred to as *MC models* were trained with spatial dropout layers after each residual block of the ResNets model. At test time, we utilized N=50 MC iterations. The dropout rates, selected empirically and supported by existing literature, were set at 0.2, 0.2, and 0.1 for ROP, knee osteoarthritis, and breast density, respectively.

### 2.4 Model training

Building upon our previous work, we employed the architectures identified as optimal for each prediction task [24]. We utilized ResNet architectures [40] selected based on initial data exploration and empirical results. All ROP models were using a ResNet18, and all knee osteoarthritis and breast density models a ResNet50. A detailed description of the training parameters can be found in S2 Text.

### 2.5 Evaluation

#### 2.5.1 Metrics.

**Ranked datasets.** The model performance was evaluated based on the ranked test data using the following three metrics. First, we computed Spearman’s rank coefficient between the rank and the predicted score. We expect a monotonic increase in the continously valued prediction scores with an increase in the expert assigned rank; hence, a Spearman coefficient of 1 corresponds to a perfect correlation. Second, we computed the agreement between the ground truth rank and the rank based on the continuous score using mean squared error (MSE) to quantify the correspondence between the predictions and ground truth. Here, the ranks were normalized to the maximum rank. Finally, the classification performance was assessed using clinically relevant AUROCs. We defined the clinically relevant classification as normal/pre-plus vs. plus for ROP, none/doubtful vs. mild/moderate/severe for knee osteoarthritis, and fatty/scattered vs. heterogeneous/dense for breast density.

**Retinopathy of prematurity.** A subset of the ROP test set had expert ratings from 1 to 9 based on the quantitative scale previously published by Taylor et al. [27]. The correspondence between the expert rating and the continuous predicted scores were measured using the MSE.

#### 2.5.2 Statistical analysis.

Metrics were bootstrapped (500 iterations) and 95% confidence intervals were evaluated for statistical analysis. Bootstrapped metrics yielding two-sided *t*-test with a p-value lower than 5% were considered statistically different.

## 3 Results

### 3.1 Predicted score compared with severity rankings

**Agreement between predicted score and severity rankings.** We first assessed how well the predicted continuous scores reflect a ranking of the images in each dataset. Retinal photographs and knee radiographs were ranked by domain experts with increasing disease severity. The mammograms were ranked with increasing density based on the quantitative continuously valued Volpara Density measurements.

The relationship between the ground truth rankings and the predicted continuous scores is presented in Fig 2. For all datasets, the multi-class models without MC dropout display horizontal plateaus around the class boundaries where the predicted score is more or less constant with increasing rank. Similar patterns can be observed for the twin and MC twin models, especially for normal ROP and knee osteoarthritis cases.

**Agreement between ranked predictions and ground truth rankings.** A linear correlation between the predicted continuous score and the consensus rank cannot be assumed as the predicted score will increase variably depending on the severity increase from a patient of rank *n* to n+1. Therefore, we used the Spearman correlation coefficient and MSE to quantify the agreement between the ground truth ranking and the ranking based on the predictions (see Table 2 and S1 Fig).

**Table 2:** Model performance overview (MEAN ± 95% CI). Bold values indicate a statistical difference (p-value<0.05) was observed between the conventional and MC dropout models. Spearman’s rank correlation coefficient and the AUROC are measured on the predicted continuous score while the MSE is measured between the normalized ground truth rank and the predicted rank generated from continuous scores. AUROC was measured between normal and pre-plus vs. plus for ROP, none and doubtful vs. mild, moderate, severe for knee osteoarthritis, and fatty and scattered vs. heterogeneous and dense for breast density.

Model	MSE ↓	Spearman ↑	clinically relevant AUROC ↑
**ROP**			
Multi-class	0.027±0.009	0.84±0.07	0.98±0.02
MC multi-class	0.010±0.003	0.94±0.02	0.99±0.01
Ordinal	0.015±0.005	0.91±0.04	0.99±0.01
MC ordinal	0.009±0.003	0.94±0.02	0.99±0.02
Regression	0.026±0.010	0.85±0.07	0.98±0.03
MC regression	0.017±0.005	0.89±0.05	0.98±0.02
Twin	0.020±0.007	0.88±0.05	0.99±0.01
MC Twin	0.013±0.004	0.92±0.03	0.98±0.02
**Knee osteoarthritis**			
Multi-class	0.023±0.008	0.86±0.06	0.97±0.02
MC multi-class	0.019±0.006	0.89±0.05	0.99±0.01
Ordinal	0.024±0.007	0.85±0.07	0.98±0.02
MC ordinal	0.022±0.007	0.86±0.06	0.99±0.02
Regression	0.023±0.009	0.86±0.06	0.99±0.01
MC regression	0.019±0.006	0.88±0.05	0.98±0.02
Twin	0.022±0.007	0.87±0.05	0.97±0.03
MC Twin	0.020±0.005	0.88±0.04	0.97±0.03
**Breast density**			
Multi-class	0.018±0.001	0.89±0.01	0.93±0.01
MC multi-class	0.016±0.001	0.90±0.01	0.94±0.01
Ordinal	0.016±0.001	0.90±0.01	0.93±0.01
MC ordinal	0.015±0.001	0.91±0.01	0.94±0.01
Regression	0.015±0.001	0.91±0.01	0.94±0.01
MC regression	0.011±0.001	0.93±0.01	0.94±0.01
Twin	0.013±0.001	0.92±0.01	0.91±0.01
MC Twin	0.012±0.001	0.93±0.01	0.92±0.01

All MC dropout models were associated with a statistically significant higher Spearman correlation coefficient and lower MSE compared to their non-MC counterparts (p-value <2.2e−4, see S2 Fig for pairwise statistical comparisons between the models). The higher Spearman correlation coefficients and lower MSE indicate that the addition of MC dropout during training and inference improves the ability of DL models to correctly rank the images based on continuous predictions. The models with the best correspondence between actual and predicted rank were MC multi-class and MC ordinal models for ROP, MC multi-class and MC regression for knee osteoarthritis, and MC regression and MC twin networks for breast density.

**Classification performance.** All MC dropout models showed slightly higher or comparable classification performance, as assessed by AUC, to their non-MC equivalent (see Table 2). The only exceptions were the twin network models for ROP and knee osteoarthritis and the regression knee osteoarthritis model. In these three cases, though statistically significant, the AUC of the MC models was only 0·01 (or less) lower the one of their non-MC equivalents. The model associated with the best classification did not necessarily correspond to the best continuous severity scores.

The following models have the overall best performance for each dataset: MC multi-class (knee osteoarthritis), MC ordinal (ROP), and MC regression (breast density).

### 3.2 Comparison of predicted ROP scores with disease severity ratings

Next, we evaluated the correspondence between the predicted scores and more detailed severity ratings generated by domain experts.

A subset of the ROP test dataset was rated by five experts on a scale from 1 to 9 instead of the standard scale from 1 to 3 [27]. This dataset allowed us to evaluate the quality of the continuous model outputs on a more granular scale than the 3-class labels on which the models were trained. Perfect continuous predictions would result in increasing disease severity scores with increasing ground severity ratings. All MC models showed a higher correspondence between the true severity ratings and predicted scores, as reflected by a lower MSE in comparison with their conventional counterparts (see Fig 3). The models predicting the experts’ ratings the most accurately are the MC multi-class and MC ordinal models.

Although twin networks showed decent correspondence between the predicted score and the ranked severity, a direct comparison with the severity ratings reveals that the predictions from these models are not well calibrated. The multi-class model without MC showed the second worst performance in this analysis. Images rated from 1 to 3 by experts mainly obtained scores near 0, which does not highlight the severity differences as perceived by human experts. Furthermore, for retinal photographs associated with a score of 4, the model predicted values on the entire spectrum, i.e., from 1 to 9, which is undesirable.

**Detection of temporal changes in disease severity.** Another important characteristic of a reliable severity score is its ability to reflect slight changes in disease severity over time. Disease evolution was quantified as the difference in the ground truth severity ratings or predicted severity scores between photographs of the same patient taken at different time points. We then compared the difference in experts’ ratings to the difference in the predicted scores using MSE (see Fig 4). Ideally, the difference in the expert’s scores should be equal to the difference in the models’ predictions. MC dropout improved the correspondence between the disease evolution as perceived by experts and predicted by the DL models for multi-class, ordinal, and twin models. Prediction differences from MC multi-class and MC ordinal models matched the severity shifts in the experts’ ratings most closely. The conventional multi-class model presents multiple outliers and is associated with the highest MSE.

### 3.3 Comparing predicted breast density scores with continuously valued ground truth breast density measurements

Lastly, we evaluated the ability of the breast density prediction models trained to accurately reflect the continuously valued Volpara Density measurements. The subset of mammograms with the Volpara Density measurements provided us with the unique opportunity to evaluate algorithms trained using ordinal labels on a continuously valued independent measurement. Therefore, unlike with the ranked score analysis presented in Sect 3.1, here we directly compared the Volpara Density measurements with the continuously valued model predictions. Ideal continuously valued predictions would correlate linearly with the Volpara Density.

We first assessed the relationship between the Volpara Density measurements and the discrete ground truth labels generated by domain experts used for training. As illustrated in the boxplot in Fig 5A, and by the Spearman correlation coefficient of 0.73, there is a high agreement between the expert-derived ground truth labels and the Volpara Density measurements.

The MC multi-class model’s predictions, both class and continuous score, are a close proxy to the volumetric breast density measurements, as seen in Fig 5B and 5C with a Spearman correlation coefficient of 0.803 (classification) and Pearson correlation coefficient of 0.91 (continuous scores). The high correlation between the continuous breast density predictions and Volpara Density measurements indicates that our model is able to generate an accurate continuous prediction while being trained on only a finite number of classes.

## 4 Discussion

The underlying continuous nature of many prediction targets for DL image analysis tasks, such as breast density and disease severity, has to be considered in the model design process. Here, we studied the capability of DL models to intrinsically learn a continuous score while being trained using discrete ordinal labels. Our experiments demonstrate that models explicitly leveraging the ordinal structure of labels consistently outperform standard multi-class classification methods, aligning with and extending prior findings from Redd et al. and Li et al. [10,22]. Additionally, we observe that incorporating MC dropout further enhances the stability and reliability of continuous score predictions. Specifically, training a conventional multi-class classification model without MC dropout does not lead to predictions that reflect the underlying continuous nature of the target variable. Approaches that model the relationship between the ordinal labels, such as ordinal classification, regression, and twin networks, provide continuous predictions that closely capture the continuity of the target variable even without the use of MC dropout. Finally, using MC dropout during training and inference increased the ability of the DL models to predict meaningful continuous scores. MC dropout multi-class classification consistently ranked among the best performing models in this study.

**Multi-class classification models.** Ignoring the ordinal relationship between the training label classes causes conventional multi-class prediction models to return predictions that are clustered around the values of the training labels. This behavior is reflected in the plateaus visible in Fig 2, and the medians in Fig 3, and S2 Fig, a lower Spearman correlation coefficient and higher MSE.

Due to the definition of the training objective, multi-class classification models are optimized to precisely predict a specific class and discouraged from predicting scores at the class boundaries. This behavior is desirable for nominal classification, where the classes should be separated as clearly as possible with minimal overlap in the feature latent space to avoid ambiguous predictions. However, the approach is not appropriate for problems with a target variable with an underlying continuous nature and explains the limited performance of the multi-class classification models to predict meaningful continuous scores.

**Twin networks.** Twin networks showed decent correspondence between the ranked severity and the predicted score (Fig 2). However, a direct comparison between the predicted score and the severity determined by domain experts (Fig 3), reveals that the predictions are not well calibrated. The predictions do not accurately reflect disease severity on a more granular scale than the labels used for model training.

Unlike the other models we evaluated, twin networks are not trained to predict a specific value, but rather to detect whether two images represent the same or different classes [41]. Therefore, they can pick up subtle differences in disease severity [10]. Here, we obtained predictions comparing the input image of interest to a pool of anchor images that are typical representations of the class corresponding to the lowest label score. While the predicted difference between the anchor images and the target images resulted in accurate ordinal predictions (Fig 2), it was not well calibrated to the underlying continuous variable, particularly at the extremes.

**MC dropout improves prediction of continuous variables.** Through the use of MC dropout, all four model types showed an improvement in the quality of the continuous scores as reflected in significantly higher Spearman correlation coefficients and lower MSE (see Table 2). MC multi-class classification networks were consistently among the highest performing models for all tasks and datasets, making them the overall top-performing models in our study.

MC dropout presents a simple way to obtain meaningful continuous predictions from models trained using ordinal labels without sacrificing and, in some cases, even significantly improving predictive performance (see Table 2). However, MC dropout comes at a higher computational cost as inference requires multiple passes of the same input image to obtain the final prediction. If the additional computational burden is a concern, ordinal classification or regression are alternatives to conventional multi-class classification models that are easy to train and provide decent continuous predictions without the use of MC dropout.

Recent work by Takezaki et al. explored an alternative approach to bridging ordinal labels and continuous severity scores by generating synthetic images at continuous severity levels using a GAN-based augmentation method [42]. While their approach is valuable for data augmentation, it does not directly learn to predict continuous scores from ordinal labels. In contrast, our work focuses on training models that directly output continuous predictions, demonstrating that meaningful continuous scores can be derived from discrete ordinal labels without the need for synthetic data.

**Limitations.** There are some limitations to this study. First, we treated the available ordinal labels as ground truth. For all three image analysis tasks analyzed here, high inter-rater variability, particularly around the decision boundaries between severity classes, have been reported [1,43–45]. It would be desirable for future work to explore the influence of noisy and biased ordinal ratings for the task of learning and predicting a continuous variable. Second, due to the latent nature of the variable of interest, for most of our analysis, we had to rely on proxy variables such as rankings and more granular expert disease severity ratings. Furthermore, other uncertainty modeling techniques, such as deep ensembles, may provide similar benefits to MC dropout dropout in terms of improving the quality of continuous score predictions. While we did not explicitly test ensembling in this study, future work should explore whether ensembling-based methods exhibit comparable improvements in recovering the continuous nature of ordinal targets. Lastly, MC dropout predictions were based on 50 samples, an empirically chosen value based on common practices and our own experience.

## Conclusion

In this work, we evaluate various model development strategies for convolutional neural networks to predict accurate continuous scores from medical images using discrete ordinal labels for model development. Our findings are particularly relevant to disease severity prediction tasks as the available labels are usually coarse and ordinal, but continuous disease severity predictions could provide crucial information that allows for earlier detection of deterioration and more personalized treatment planning.

## Code availability

The code used to train the models can be found at https://github.com/andreanne-lemay/gray_zone_assessment.

## Supporting information

S1 Fig Correspondence between model predicted rank and true severity rank. For each model, the Pearson correlation coefficient (*r*) is displayed and indicates the strength of the linear correlation where 1 is a perfectly positive linear correlation and -1 is a perfectly negative linear correlation.
(TIFF)

S2 FigPair-wise statistical comparisons for MSE, Spearman, and AUC metrics (metric - dataset).A: MSE - Knee osteoarthritis; B: MSE - Breast density; C: Spearman correlation coefficient - Knee osteoarthritis; D: AUROC - ROP (100 ranked cases); E: AUROC - Knee osteoarthritis; F: AUROC - Breast density. Each pair of models (MC and non-MC multi-class, ordinal, regression, twin) was compared for each metric on a given test set. The box plots on the left side display the value range obtained through 500 bootstraps. The grid on the right side includes the 28 pair-wise comparisons. * means that a statistical difference (p−value<0.05 on a two-sided t-test) was reached, while a black square indicates no statistical differences. Only metrics where at least one pair had no statistical difference are presented.(TIFF)

S3 FigCorrespondence between predicted and consensus ROP severity on an out-of-distribution test set.The rater score is obtained from a single rater. The predicted scores from multi-class, ordinal, and regression models that were trained to predict values from 0 to 2 were scaled and shifted to match the 1–9 range ($score_{rescaled}=score_{model}\times 2+1$). Twin networks predict values from 0 to infinity and is not fully bounded. The twin network scores were hence only shifted by 1 ($score_{rescaled}=score_{Twin}+1$). All MSE measurements reported in this figure are statistically different (p−value<0.05).(TIFF)

S1 Text Summary of dataset label distributions. Overview of label frequencies across the three prediction tasks: Retinopathy of prematurity disease severity in retinal photographs, knee osteoarthritis severity in radiographs, and breast density in mammograms. For each dataset, the total number of images and the distribution across all ordinal categories are listed.
(DOCX)

S2 Text Model architecture and training specifications. Overview of training parameters for the ResNet18 and ResNet50 models used across the Retinopathy of prematurity, knee osteoarthritis, and breast density prediction tasks, including batch sizes, learning rates, training duration, validation-based model selection, class balancing strategies, and applied data augmentations.
(DOCX)
