# Supplementary figures and images for: Leveraging deep learning to infer continuous predictions from ordinal labels in medical imaging
Source: PLOS Digit Health. 2026 Apr 17;5(4):e0001248. doi: 10.1371/journal.pdig.0001248 (PMC13089899; doi:10.1371/journal.pdig.0001248)

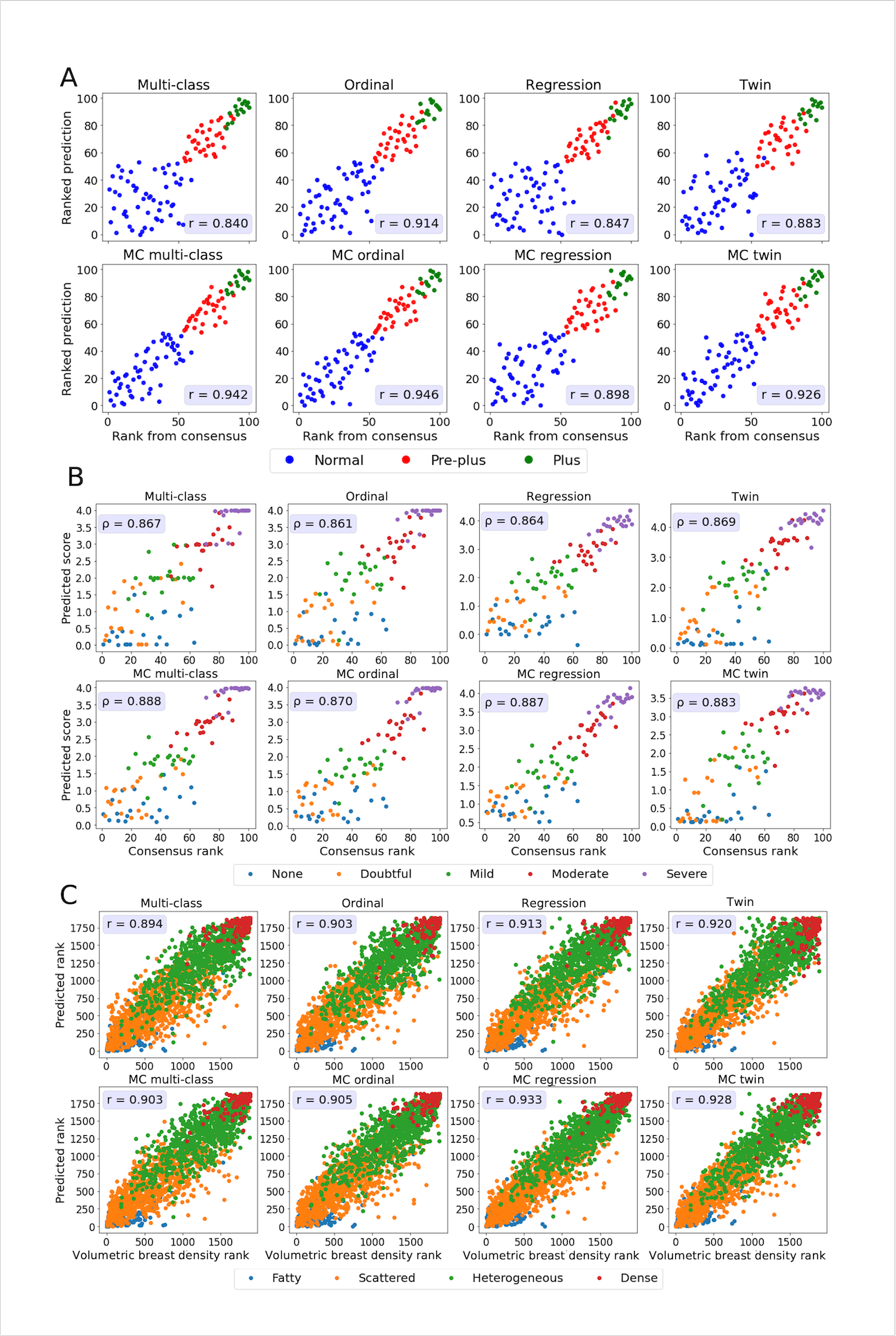

Supplement: S1 Fig — For each model, the Pearson correlation coefficient (r) is displayed and indicates the strength of the linear correlation where 1 is a perfectly positive linear correlation and -1 is a perfectly negative linear correlation. (TIFF) [file pdig.0001248.s001.tiff]

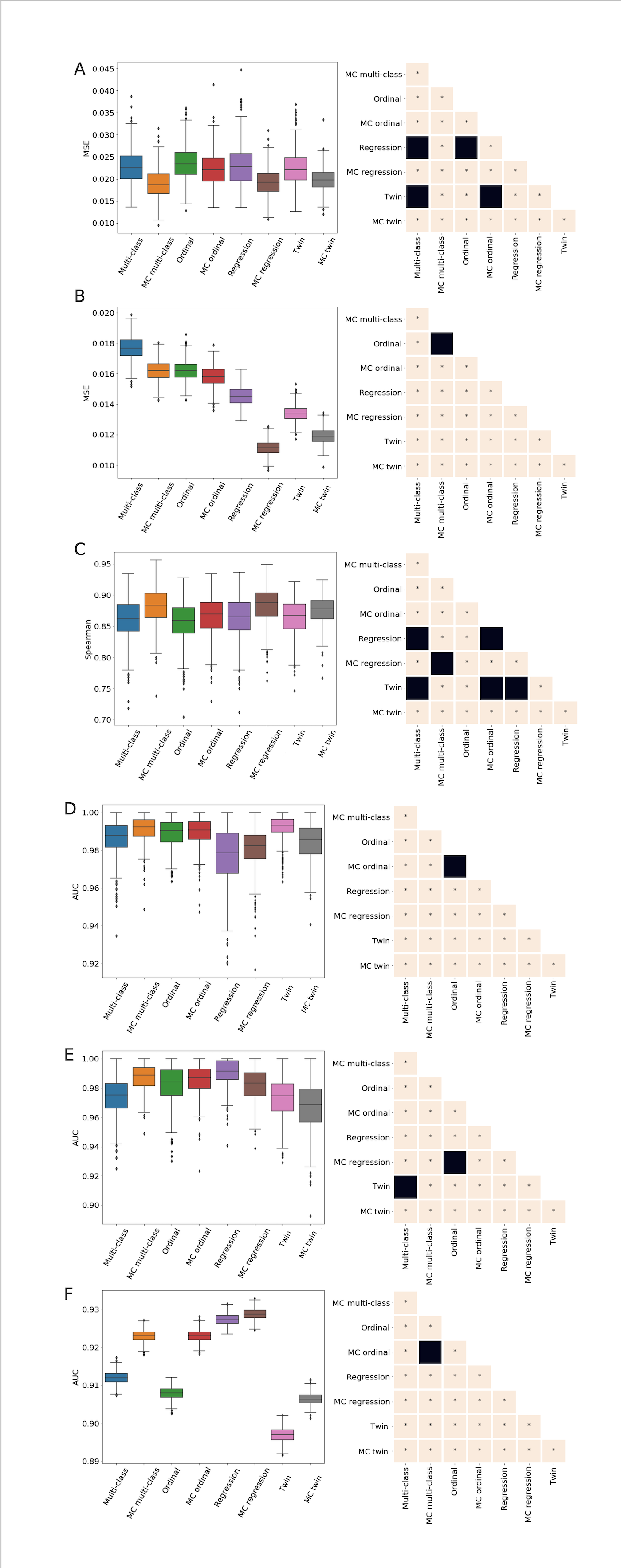

Supplement: S2 Fig — A: MSE - Knee osteoarthritis; B: MSE - Breast density; C: Spearman correlation coefficient - Knee osteoarthritis; D: AUROC - ROP (100 ranked cases); E: AUROC - Knee osteoarthritis; F: AUROC - Breast density. Each pair of models (MC and non-MC multi-class, ordinal, regression, twin) was compared for each metric on a given test set. The box plots on the left side display the value range obtained through 500 bootstraps. The grid on the right side includes the 28 pair-wise comparisons. * means that a statistical difference (p−value<0.05 on a two-sided t-test) was reached, while a black square indicates no statistical differences. Only metrics where at least one pair had no statistical difference are presented. (TIFF) [file pdig.0001248.s002.tiff]

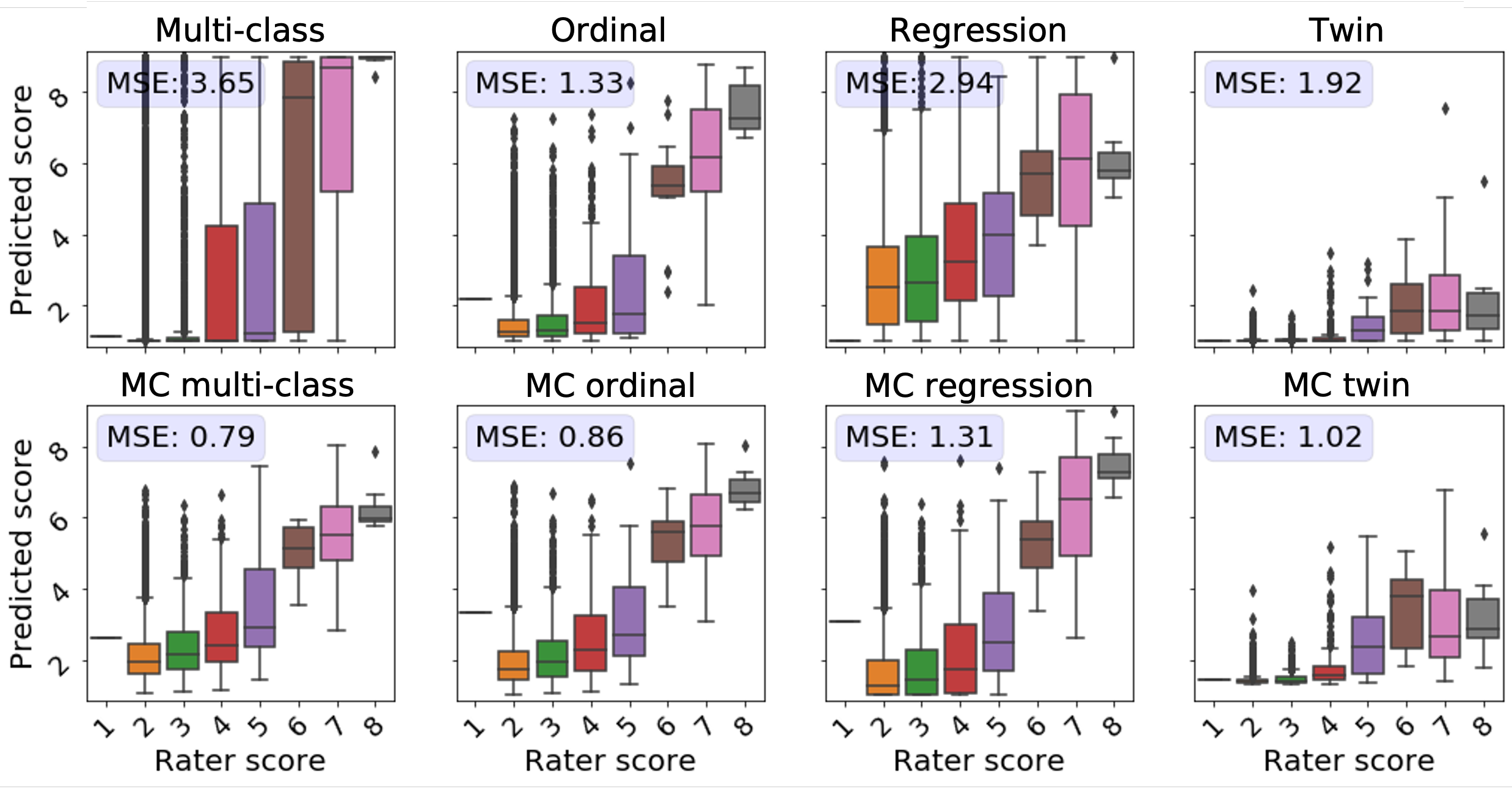

Supplement: S3 Fig — The rater score is obtained from a single rater. The predicted scores from multi-class, ordinal, and regression models that were trained to predict values from 0 to 2 were scaled and shifted to match the 1–9 range (scorerescaled=scoremodel×2+1). Twin networks predict values from 0 to infinity and is not fully bounded. The twin network scores were hence only shifted by 1 (scorerescaled=scoreTwin+1). All MSE measurements reported in this figure are statistically different (p−value<0.05). (TIFF) [file pdig.0001248.s003.tiff]
