# Supplementary material for: Leveraging deep learning to infer continuous predictions from ordinal labels in medical imaging
Source: PLOS Digit Health. 2026 Apr 17;5(4):e0001248. doi: 10.1371/journal.pdig.0001248 (PMC13089899; doi:10.1371/journal.pdig.0001248)
Supplement: S1 Text — Overview of label frequencies across the three prediction tasks: Retinopathy of prematurity disease severity in retinal photographs, knee osteoarthritis severity in radiographs, and breast density in mammograms. For each dataset, the total number of images and the distribution across all ordinal categories are listed. (DOCX) [file pdig.0001248.s004.docx]

S1 Text - Dataset label distributions

List of label distributions for each dataset.

**Retinopathy of prematurity**

Dataset size: 5511 images

- Normal: 4535 images (82.3%)
- Pre-plus disease: 804 images (14.6%)
- Plus disease: 172 images (3.1%)

**Knee osteoarthritis (OA)**

Dataset size: 14173 images

- No OA (KL 0): 5793 images (40.9%)
- Doubtful OA (KL 1): 2156 images (15.2%)
- Mild OA (KL 2): 2355 images (16.6%)
- Moderate OA (KL 3): 2604 images (18.4%)
- Severe OA (KL 4): 1265 images (8.9%)

**Breast density**

Dataset size: 108230 images

- Fatty: 12428 images (11.5%)
- Scattered: 47909 images (44.2%)
- Heterogeneously dense: 41325 images (38.2%)
- Dense: 6568 images (6.1%)
