# Supplementary material for: Leveraging deep learning to infer continuous predictions from ordinal labels in medical imaging
Source: PLOS Digit Health. 2026 Apr 17;5(4):e0001248. doi: 10.1371/journal.pdig.0001248 (PMC13089899; doi:10.1371/journal.pdig.0001248)
Supplement: S2 Text — Overview of training parameters for the ResNet18 and ResNet50 models used across the Retinopathy of prematurity, knee osteoarthritis, and breast density prediction tasks, including batch sizes, learning rates, training duration, validation-based model selection, class balancing strategies, and applied data augmentations. (DOCX) [file pdig.0001248.s005.docx]

S2 Text - Model training parameters

**Retinopathy of Prematurity**

ROP models had a ResNet18 architecture and were trained with a batch size of 24 and a learning rate of 1e-4 for 25 epochs, and the best model was selected using the highest accuracy on the validation set. Balanced class sampling mitigated the class imbalance during training. Data augmentation consisted of random rotation of ±15 degrees with a probability of 0.5, random flips with a probability of 0.5, and random zooms of 0.9 to 1.1 with a probability of 0.5.

**Knee osteoarthritis**

ResNet50 architecture was selected for the knee osteoarthritis model and was trained with the following parameters: batch size of 16, learning rate of 5e-6, 75 epochs. The final model was chosen based on the best loss value on the validation set. The data sampler used balanced weights during training to help with data imbalance. Images were randomly rotated of ±15 degrees with a probability of 0.5 and randomly flipped with a probability of 0.5 as data augmentation.

**Breast density**

Breast density models were trained with a ResNet50 architecture for 75 epochs by batches of 8 with a learning rate of 5e-5. The best model was selected using the best loss score on the validation set. The same data augmentation as the knee osteoarthritis model was applied for breast density.
